# Mangiferin alleviates metabolic-associated fatty liver disease by modulating gut microbiota and FXR signaling pathway to regulate bile acid metabolism

**DOI:** 10.3389/fphar.2026.1844895

**Published:** 2026-06-16

**Authors:** Linlin Zheng, Qiyan Xie, Si Yao, Mimi Chen, Wenwen Zhao, Ruodi Li, Jinke Cheng, Yong Zhang

**Affiliations:** 1 Key Laboratory of Tropical Translational Medicine of Ministry of Education, School of Basic Medicine and Life Sciences, Hainan Medical University, Haikou, China; 2 Hainan Academy of Medical Sciences, Hainan Medical University, Haikou, China

**Keywords:** MAN, MAFLD, BAs, gut microbiota, FXR

## Abstract

**Background:**

Metabolic-associated fatty liver disease (MAFLD) is characterized by excessive hepatic lipid accumulation, with limited safe and effective therapeutic options currently available. Previous studies have demonstrated that mangiferin (MAN) alleviates nonalcoholic fatty liver disease via modulation of the AMPK and NLRP3 signaling pathways. However, there are no reports to date investigating whether MAN exerts anti-MAFLD effects by regulating bile acid (BAs) metabolism and its underlying molecular mechanisms.

**Methods:**

In the present study, the anti-MAFLD effects of MAN were systematically investigated using a high-fat diet (HFD)-induced MAFLD mouse model. We evaluated the therapeutic mechanisms of MAN specifically from the perspectives of BAs metabolism regulated by the farnesoid X receptor (FXR) signaling pathway and the modulation of the gut microbiota, utilizing 16S rRNA sequencing and molecular docking analyses.

**Results:**

MAN treatment (100 mg/kg) significantly ameliorated glucose and lipid metabolic disorders, as well as hepatic lipid accumulation in the HFD-induced MAFLD mice, which was accompanied by marked alterations in the BAs metabolic profile. Mechanistically, MAN activated the FXR signaling pathway, and molecular docking analysis predicted stable interactions with key FXR residues (Y365, M369, and Y373). Furthermore, 16S rRNA sequencing revealed that MAN significantly decreased the relative abundance of *Lactobacillus* and *Limosilactobacillus*, which were positively correlated with abnormal BAs (including 6,7-DKLCA, ILCA, GHDCA, and GUDCA), dyslipidemia, and liver injury markers. In contrast, MAN increased the relative abundance of *Faecalibaculum*, which was negatively correlated with these BAs and associated with an improved metabolic status.

**Conclusion:**

MAN exerts anti-MAFLD effects through dual mechanisms: direct activation of the FXR signaling pathway to regulate BAs homeostasis and indirect modulation of the gut microbiota to influence BAs metabolism. These findings highlight the therapeutic potential of MAN and provide new insights into natural product-based strategies for MAFLD treatment.

## 1 Introduction

Metabolic-associated fatty liver disease (MAFLD) refers to fatty liver disease linked to systemic metabolic disorders, with its pathological changes involving interconnected processes such as glucose-lipid metabolic dysregulation, inflammation, and liver fibrosis (Huang X. et al., 2023). Relevant surveys indicate that the global incidence of MAFLD reached 25% in 2021 and is continuously increasing (Thomas et al., 2022). However, effective pharmacological therapies for MAFLD remain unavailable, underscoring the urgent need to develop potent and targeted therapeutic agents for its management.

Bile acids (BAs) are synthesized in the liver through a series of enzymatic reactions and stored in the gallbladder, where they play a crucial role in solubilizing lipophilic substances in the small intestine (Russell, 2009; Chiang, 2013). Farnesoid X receptor (FXR), also known as the bile acid receptor, is highly expressed in the liver and intestine of mammals. In mice, FXR directly activates the transcription of genes encoding small heterodimer partner (SHP) in the liver and fibroblast growth factor 15 (FGF15; FGF19 in humans) in the intestine. Both SHP and FGF15 inhibit the expression of enzymes involved in hepatic bile acid synthesis (Goodwin et al., 2000; Kong et al., 2012). A large body of animal studies and clinical research has demonstrated that serum and hepatic bile acid levels are significantly elevated in patients with MAFLD, with a severe imbalance in the composition of bile acid species (Zhu et al., 2018; Molinaro et al., 2018; Liu et al., 2024). Furthermore, the onset and progression of MAFLD are often accompanied by notable gut microbiota dysbiosis, which further impacts bile acid metabolism and FXR signaling, exacerbating hepatic lipid accumulation and inflammatory responses (Wang et al., 2025). As a core regulator of bile acid metabolism, FXR plays a pivotal role in the pathogenesis and progression of MAFLD through multiple mechanisms. Therefore, targeting the FXR signaling pathway to regulate bile acid metabolism and restore gut microbiota balance holds promising therapeutic potential for the treatment of MAFLD (Dawson and Karpen, 2015).

In recent years, the role of the gut microbiota in MAFLD has attracted increasing attention. Accumulating evidence indicates that gut microorganisms can mediate bile acid deconjugation through bile salt hydrolase (BSH) activity and participate in the generation of secondary bile acids, thereby altering bile acid composition and bioactivity (Collins et al., 2023). Bile acids are not only essential for lipid digestion but also function as key signaling molecules regulating metabolic homeostasis, and alterations in their composition are closely associated with the onset and progression of MAFLD. Therefore, the “gut microbiota–bile acid metabolism” axis is considered a critical link connecting intestinal microecology with host metabolism. From a clinical perspective, microbiota-targeted interventions, including probiotics, prebiotics, and fecal microbiota transplantation (FMT), have been explored for the treatment of MAFLD. Studies have shown that supplementation with specific probiotics can improve liver function parameters and lipid profiles, and partially alleviate hepatic steatosis (Ma et al., 2013; Asghari et al., 2025). In addition, FMT, as a direct approach to modulate gut microbial composition, has demonstrated potential in improving insulin resistance and hepatic metabolic dysfunction in certain clinical trials (Abenavoli et al., 2022). Collectively, these findings further support the pivotal role of the gut microbiota in the therapeutic intervention of MAFLD.

Mangiferin (MAN), also known as iriflophenone 3-C-glucoside, is a structurally unique C-glycoside compound. It is primarily found in various higher plants and mango fruits, including the peel and seed, as well as in mango tree tissues such as the leaves and branches (Imran et al., 2017; Muruganandan et al., 2005; Sánchez et al., 2000). Previous studies have shown that MAN exhibits a wide range of pharmacological activities, including anti-cancer, anti-inflammatory, anti-diabetic, cardiovascular protective, antioxidant, neuroprotective, anti-hyperlipidemic, and metabolic regulatory effects on glucose and lipids (Peng et al., 2015; Zhang et al., 2014; Fu et al., 2014; Carpene et al., 2015; Kavitha et al., 2020; Zhou et al., 2015). In addition, accumulating evidence suggests that MAN may exert its pharmacological effects by modulating the gut microbiota (Bunt et al., 2025). Based on these known biological activities, this study further investigates the role of MAN in a high-fat diet (HFD)-induced MAFLD model and explores its underlying mechanisms, aiming to provide new theoretical insights and potential therapeutic strategies for the prevention and treatment of MAFLD.

## 2 Materials and methods

### 2.1 Materials

Mangiferin (B20837) was provided by Yuanye Bio-Technology (Shanghai, China). GW4064 (HY-50108) was purchased from MCE (USA). HepG2 cells (CL0103) and HT29 cells (CL-0118) were obtained from Procell Life Science and Technology (Wuhan, China). DMEM (11995065), DMEM/F12 (11330032), fetal bovine serum (FBS, 16000044), NEAA (11140050) and Penicillin-Streptomycin (P/S) (15140122) were supplied by Gibco (USA). TB Green Premix Ex Taq II (Tli RHaseH Plus) (RR820A) and PrimeScript RT Master Mix (Perfect Real Time) (RR036A) were supplied by TaKaRa (Japan). A high-fat diet (HFD, 60 kcal% Fat) (D12492) was purchased from Future Biotech (Beijing, China). Total Cholesterol (CHO) Assay Kit (A111-1-1), Aspartate Aminotransferase (AST) Assay Kit (010-2-1), Alanine Aminotransferase (ALT) Assay Kit (C009-2-1), Triglyceride (TG) Assay Kit (A110-1-1), Low-Density Lipoprotein Cholesterol (LDL-C) Assay Kit (A113-1-1), High-Density Lipoprotein Cholesterol (HDL-C) Assay Kit (A112-1-1), and Total Bile Acid (TBA) Assay Kit (E003-2-1) were provided by Njjcbio (Nanjing, China). Human Fibroblast Growth Factor 19 (FGF19) ELISA Kit (JL19321), Mouse Fibroblast Growth Factor 15 (FGF15) ELISA Kit (JL49232) and Total Bile Acid (TBA) Content Assay Kit (Enzymatic Cycling Colorimetric Method) (JL-T1373) were obtained from JONLNBIO (Shanghai, China). Pierce BCA Protein Assay Kit (23225) was supplied by Thermo Fisher Scientific (United States). FXR/NR1H4 Antibody (72105) was purchased from CST (United States). FGF15 Antibody (ab229630) was supplied by Abcam (UK). FGF19 Antibody (sc-390621) and SHP Antibody (SC-271511) was purchased from Santa Cruz Biotechnology (United States). CYP7A1 Antibody (GB150112) and β-Actin Antibody (GB150003) was provided by Servicebio (Wuhan, China). Anti-rabbit IgG, HRP-linked Antibody (7074) and Anti-mouse IgG, HRP-linked Antibody (7076) were supplied by CST (United States).

### 2.2 Cell culture

HepG2 and HT29 cells were cultured at 37 °C with 5% CO_2_. HepG2 cells were maintained in DMEM medium supplemented with 10% FBS, 1 × NEAA and 1% P/S, while HT29 cells were cultured in DMEM/F12 medium containing 5% FBS and 1% P/S. HepG2 and HT29 cells were seeded into well plates, and the test drugs were added after 24 h of incubation. Proteins and RNA were then extracted separately.

### 2.3 FGF19 detection

The concentration of FGF19 in the cell supernatant was measured using an FGF19 ELISA Kit following the manufacturer’s instructions. The cell culture supernatant was centrifuged at 1000×g for 20 min, and the supernatant was collected for ELISA detection. The optical density (OD) value of each well was determined at a wavelength of 450 nm using a microplate reader.

### 2.4 Animals and experimental design

Male C57BL/6J mice (5 weeks old) were purchased from GemPharmatech Co., Ltd (Jiangsu, China). All animal experiments were performed at the Animal Research Center of Hainan Medical University in accordance with the guidelines of the National Institutes of Health (NIH) and approved by the Medical Ethics Committee of Hainan Medical University. Mice were housed in a controlled environment at the Animal Research Center of Hainan Medical University, with a temperature of 25 °C ± 0.5 °C, humidity of 55% ± 5%, and a 12-h light/dark cycle.

After a 3-day acclimatization period, all mice were randomly divided into 4 groups (n = 6 per group): normal control group, MAFLD model group, mangiferin low-dose group (hereafter as MAN-L, 25 mg/kg/day), and mangiferin high-dose group (hereafter as MAN-H, 100 mg/kg/day) (Yong et al., 2021).

Mice in the normal control group were fed a standard chow diet; mice in the MAFLD model group were fed a high-fat diet (HFD); mice in the MAN-L group were fed HFD and administered 25 mg/kg/day MAN via intragastric gavage (i.g.); mice in the MAN-H group were fed HFD and administered 100 mg/kg/day MAN via i.g. Mice in the normal control group and MAFLD model group were given an equal volume of normal saline. Food intake and body weight were recorded weekly. After 16 weeks of drug intervention, blood samples were collected via orbital sinus puncture for serum analysis, and tissue samples were harvested for further studies.

### 2.5 Oral glucose tolerance test and insulin tolerance test

On the 14th week after MAN, after overnight 8 h fasting, all animals were subjected to an oral glucose tolerance test (OGTT) using 2 g/kg glucose (p.o.). Blood samples were collected from the tail vein at 0, 50, 100, 150, and 200 min after glucose administration. On the 14 weeks after MAN treatment, after overnight 6 h fasting, all animals were subjected to an insulin tolerance test (ITT) using 0.5 U/kg insulin (i.p.). Blood samples were collected from tail at 0, 30, 60, and 90 min after insulin administration.

### 2.6 Serum biochemistry analysis

After 16 weeks of treatment, blood samples were collected from mice following overnight fast. The blood samples were left at room temperature for 4 h and subsequently centrifuged at 3,500 rpm for 15 min to isolate serum. TG, TC, LDL-c, HDL-c, ALT, AST, TBA and FGF15 were measured in accordance with the manufacturer’s instructions provided with each corresponding assay kit.

### 2.7 Hepatic TBA determination

The concentration of TBA in liver tissues was measured using the TBA Content Assay Kit (Enzymatic Cycling Colorimetric Method) in strict accordance with the manufacturer’s instructions. Liver tissue homogenates were centrifuged at 12,000 rpm for 10 min, and the resulting supernatants were collected for subsequent ELISA analysis. The absorbance was measured at a wavelength of 405 nm. Finally, the hepatic TBA levels were quantified by normalizing against the protein concentrations determined via the BCA assay.

### 2.8 Hematoxylin and eosin (H&E) staining and Oil Red O staining

Upon euthanasia, liver tissues were promptly collected, rinsed with normal saline, and cut into pieces. The samples were fixed in 10% formalin for 48 h to ensure full fixation, followed by paraffin embedding and sectioning into 5 μm slices. Sections were stained with H&E for histological evaluation and with Oil Red O to assess lipid accumulation. Stained sections were examined under a light microscope, and images were acquired for further analysis.

### 2.9 qRT-PCR

RNA concentration was determined spectrophotometrically. Reverse transcription was performed with PrimerScript RT Master Mix Kit to generate cDNA, and quantitative PCR was conducted. The relative expression of target genes were calculated by the 2^−ΔΔCt^ method. All primers used in this study, synthesized by Sangon Biotech (Shanghai, China) Co., Ltd., are listed in Table 1.

**TABLE 1:** qRT-PCR primer sequences.

Gene	Primer sequence (5′-3′)
Human *FXR*	F: ACT​TCC​GTC​TGG​GCA​TTC​TGA​C
	R: GCT​GTA​AGC​AGA​GCA​TAC​TCC​TC
Human *FGF19*	F: CGG​AGG​AAG​ACT​GTG​CTT​TCG
	R: CTC​GGA​TCG​GTA​CAC​ATT​GTA​G
Human *β-ACTIN*	F: GAA​GAT​CAA​GAT​CAT​TGC​TCC​TC
	R: ATC​CAC​ATC​TGC​TGG​AAG​G
Mouse *Shp*	F: TGG​GTC​CCA​AGG​AGT​ATG​C
	R: GCT​CCA​AGA​CTT​CAC​ACA​GTG
Mouse *Cyp7a1*	F: CAA​GAA​CCT​GTA​CAT​GAG​GGA​C
	R: CAC​TTC​TTC​AGA​GGC​TGC​TTT​C
Mouse *Fxr*	F: GCT​TGA​TGT​GCT​ACA​AAA​GCT​G
	R: CGT​GGT​GAT​GGT​TGA​ATG​TCC
Mouse *Fgf15*	F: ATG​GCG​AGA​AAG​TGG​AAC​GG
	R: GGA​CCA​GCG​GAG​TAC​AGG​T
Mouse *β-Actin*	F: GTG​ACG​TTG​ACA​TCC​GTA​AAG​A
	R: GCC​GGA​CTC​ATC​GTA​CTC​C

### 2.10 Western blot assay

Protein concentration was determined using a BCA assay, adjusted, mixed with 5× SDS-PAGE loading buffer, boiled at 100 °C for 10 min, and either used immediately or stored at −20 °C. Proteins were separated by SDS-PAGE (40 V for stacking gel, 100 V for separating gel) and transferred to PVDF membranes (400 mA, 4 °C, 40 min). Membranes were blocked with 5% non-fat milk in TBST at 4 °C overnight, washed with TBST for 5 min, incubated with primary antibodies at room temperature (RT) for 2 h, washed with TBST three times (10 min each), incubated with secondary antibodies at RT for 1.5 h, and washed again with TBST three times (10 min each). Detection was performed using ECL, and imaging was conducted with a Tanon system. Band intensities were analyzed using ImageJ software.

### 2.11 *Fxr* knock down by shRNA

Lipofectamine 3000 (L3000001; Thermo Fisher, United States) was used to transfect 293T cells for lentiviral packaging. pSPAX2, pMD2. G, and the target vector were co-transfected at a ratio of 3:3:2. After 48–72 h, GFP expression was observed under a fluorescence microscope. Viral supernatants were collected, filtered through a 0.45 μm PES filter, concentrated by centrifugation at 12,000×g, and stored at −80 °C. Next, HT29 cells were seeded in 6-well plates at a density of 5.0 × 10^4^ cells/mL. After 24 h, cells were infected with *shFxr* lentivirus, and the regular medium was replaced with medium containing 5 μg/mL puromycin for culture. GFP expression was continuously monitored, and a stable *shFxr* cell line was finally obtained.

### 2.12 MicroScale thermophoresis assay (MST)

The MST assay was outsourced to TargetMol (Shanghai, China). The experimental process included protein labeling and fluorescence detection. Proteins were labeled using the RED-NHS Protein Labeling Kit (MO-L011; NanoTemper, Germany). GW4064 and MAN were serially two-fold diluted into 16 gradients in reaction buffer (50 mM HEPES buffer [pH = 7.4] containing 0.05% Tween 20), mixed in equal volumes, and incubated at room temperature for 20 min. The mixture samples were loaded into capillaries (Monolith NT.115 Capillary), and thermophoresis signals were measured using the Monolith NT.115 following the manufacturer’s standard protocol. The dissociation constant (Kd) values were analyzed using NanoTemper Analysis Software.

### 2.13 Cellular thermal shift assay (CETSA)

CETSA was performed as previously reported (Jafari et al., 2014; Martinez Molina et al., 2013). HT29 cells were equally divided into two aliquots and incubated with 50 μM MAN (dissolved in DMSO) or DMSO alone at 37 °C for 2 h. Cells were then collected for lysis, and each lysate was split into smaller aliquots (100 μL), which were individually heated at different temperatures (37 °C–75 °C) for 5 min using a thermal cycler. Subsequently, the aliquots were rapidly frozen in liquid nitrogen and immediately thawed in a 25 °C thermal cycler for 10 s, and this freeze-thaw cycle was repeated three times. Samples were collected by high-speed centrifugation at 21,000×g for 30 min at 4, followed by Western blot analysis.

### 2.14 Molecular docking

Structural details of the relevant target proteins were retrieved from the PDB database (PDB ID: 1OSH). The protein structure was refined using PyMOL-2.1.0 software, followed by hydrogen atom addition and charge assignment using AutoDockTools-1.5.6. Visualization and analysis of the docking results were conducted using PyMOL and Discovery Studio 2019 Client software.

### 2.15 Bile acid analysis

Bile acid metabolomic profiling of samples was conducted by Wuhan MetWare Biotechnology Co., Ltd. An absolute quantification method based on bile acid standards was employed in this study. Quantification of BAs in samples was performed using an ultra-high performance liquid chromatography-tandem triple quadrupole mass spectrometer (UPLC-TQMS, Waters). Briefly, samples were subjected to preprocessing (protein precipitation, centrifugation, and filtration). Separated BAs were achieved via gradient elution in UPLC. The specific gradient elution program for mobile phases A/B was set as follows: 0 min, 95:5 (v/v); 0.5 min, 60:40 (v/v); 4.5 min, 50:50 (v/v); 7.5 min, 25:75 (v/v); 10 min, 5:95 (v/v); and 12.0 min, 95:5 (v/v). This was followed by detection using TQMS operated in electrospray ionization-multiple reaction monitoring (ESI-MRM) mode. Concentrations of individual BAs were calculated using the internal standard method and calibration curves. For subsequent metabolomic statistical analyses, significantly regulated metabolites between groups were determined by Variable Importance in Projection (VIP) scores and absolute Log2FC (fold change). VIP values were extracted from orthogonal partial least squares-discriminant analysis (OPLS-DA) results, generated using the R package MetaboAnalystR, which also provided score and permutation plots. Prior to OPLS-DA, the data were log-transformed (log2) and mean-centered. To avoid overfitting, a permutation test (200 permutations) was performed. Furthermore, identified metabolites were annotated using the KEGG compound database (http://www.kegg.jp/kegg/compound/) and subsequently mapped to the KEGG Pathway database (http://www.kegg.jp/kegg/pathway.html). Pathways containing significantly regulated metabolites were then subjected to metabolite sets enrichment analysis (MSEA), with significance determined by hypergeometric test *p*-values.

### 2.16 16S rDNA sequencing

Fresh fecal samples from mice were collected and immediately stored at −80 °C until analysis. Total microbial DNA was extracted from the fecal samples using a commercial DNA extraction kit. The V3-V4 hypervariable regions of the bacterial 16S rRNA gene were amplified using specific primers with barcode sequences to distinguish different samples within the same library. Sequencing libraries were constructed using the TruSeq Nano DNA LT Sample Preparation Kit and sequenced on the Illumina NovaSeq 6000 platform. The sequencing and initial bioinformatic analyses were performed by MetWare Biotechnology Co., Ltd (Wuhan, China). Raw sequencing data were subjected to quality filtering using the QIIME2 platform, followed by systematic statistical analysis of the microbial communities.

Regarding the statistical analysis of the data: First, the Shannon and Simpson indices were calculated to evaluate the α-diversity of the communities, and a one-way analysis of variance (ANOVA) was employed to test the significance of differences among the groups. Secondly, to evaluate β-diversity, which reflects differences in community structure, we performed principal component analysis (PCA) and phylogenetic clustering analysis. Furthermore, a distance matrix-based permutational multivariate analysis of variance (PERMANOVA/ADONIS), with the number of permutations set to 999, was applied to rigorously assess the significance of structural changes in the microbial communities among the groups.

### 2.17 Data analysis

Experimental data are expressed as the mean ± standard deviation (SD). One-way analysis of variance (ANOVA) was performed using GraphPad Prism 8.0 software, followed by Tukey’s multiple comparisons test for *post hoc* analysis. ^*^ *p* < 0.05, ^**^ *p* < 0.01, ^***^ *p* < 0.001, ^****^ *p* < 0.0001.

## 3 Results

### 3.1 Effects of MAN on MAFLD mice

**FIGURE 1:**
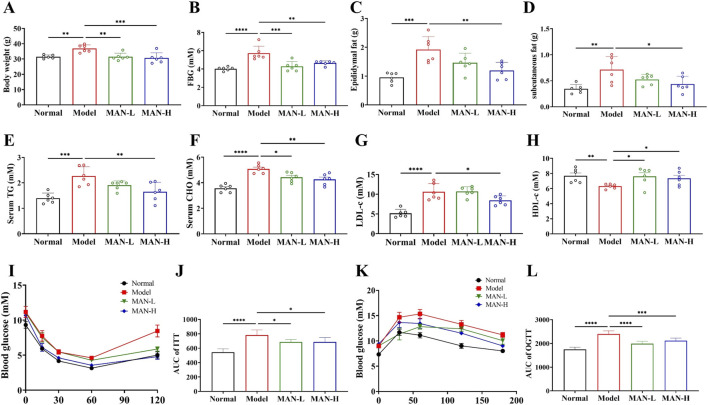
MAN ameliorates glucose and lipid metabolism in MAFLD mice. **(A)** Body weight. **(B)** Fasting blood glucose. **(C)** Epididymal fat weight. **(D)** Subcutaneous fat weight. **(E)** Serum TG levels. **(F)** Serum TC levels. **(G)** Serum LDL-C levels. **(H)** Serum HDL-C levels **(I)** ITT. **(J)** Area under of ITT. **(K)** OGTT. **(L)** Area under of OGTT. Data are expressed as the mean ± SD. ^*^ *p* < 0.05, ^**^ *p* < 0.01, ^***^ *p* < 0.001, ^****^ *p* < 0.0001.

Compared with the normal group, HFD-induced MAFLD mice exhibited a significant increase in body weight (Figure 1A), epididymal fat (Figure 1C), and subcutaneous fat (Figure 1D), while MAN intervention markedly reduced weights. Additionally, fasting blood glucose (FBG) levels were significantly elevated in HFD-induced MAFLD mice, which were notably decreased following MAN treatment (Figure 1B). Concurrently, MAFLD mice showed significantly increased serum levels of TG, TC, and LDL-C, as well as a significant decrease in HDL-C, indicating typical hyperlipidemia (Figures 1E–H). After intervention with 100 mg/kg MAN, the serum levels of TC, TG, and LDL-C in MAFLD mice were significantly reduced, while HDL-C level was increased (Figures 1E–H), further demonstrating the role of MAN in ameliorating lipid metabolism disorders. Moreover, ITT and OGTT were performed to evaluate the effects of MAN on insulin sensitivity and glucose tolerance in MAFLD mice. The results showed that MAN at a dose of 25 mg/kg significantly improved insulin sensitivity (Figures 1I,J) and glucose tolerance (Figures 1K,L) in MAFLD mice. Collectively, these findings indicate that MAN effectively ameliorates glucose and lipid metabolism disorders in MAFLD mice.

**FIGURE 2:**
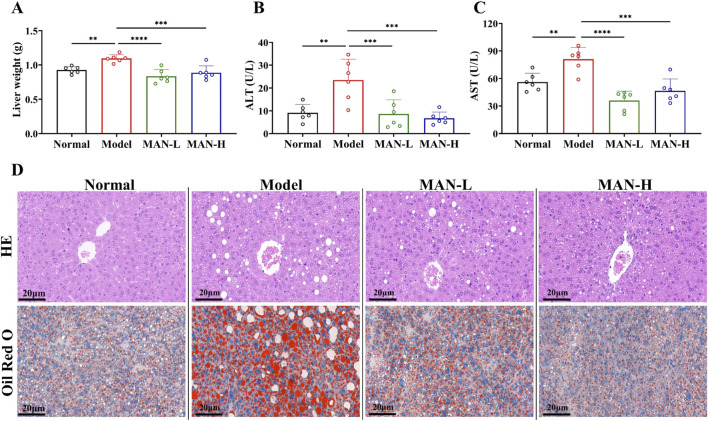
MAN alleviates liver function and lipid accumulation in MAFLD mice. **(A)** Liver weight. **(B)** Serum levels of ALT. **(C)** Serum levels of AST. **(D)** Oil Red O Staining and H&E Staining; scale bar = 20 μm. Data are expressed as the mean ± SD. ^*^ *p* < 0.05, ^**^ *p* < 0.01, ^***^ *p* < 0.001, ^****^ *p* < 0.0001.

Furthermore, we examined the therapeutic effects of MAN on liver function impairment and hepatic lipid accumulation in MAFLD mice. We found that the liver weight of mice in the MAFLD model group was significantly higher than that in the normal group, while MAN intervention significantly reduced liver weight in MAFLD mice (Figure 2A). Additionally, MAFLD mice exhibited obvious liver injury, as evidenced by elevated serum levels of ALT and AST (Figures 2B,C). After intervention with 25 mg/kg MAN, ALT and AST levels were significantly decreased, indicating that MAN can effectively alleviate liver injury in MAFLD mice. H&E and Oil Red O staining revealed that MAFLD mice showed obvious lipid droplet accumulation in liver tissues, which was significantly alleviated by MAN intervention (Figure 2D). In summary, MAN effectively mitigates MAFLD-related liver injury and metabolic abnormalities by improving liver function and reducing hepatic lipid accumulation. These results further illustrate the potential of MAN in the treatment of metabolic diseases.

### 3.2 Effects of MAN on bile acids

**FIGURE 3:**
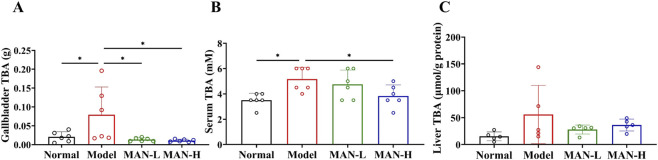
MAN affects the bile acid levels in MAFLD mice. **(A)** TBA levels in the gallbladder. **(B)** Serum TBA levels. **(C)** Hepatic TBA levels. Data are expressed as the mean ± SD. ^*^ *p* < 0.05, ^**^ *p* < 0.01, ^***^ *p* < 0.001, ^****^ *p* < 0.0001.

To further elucidate the underlying metabolic mechanisms by which MAN exerts its protective effects against MAFLD, we quantified BA levels in the gallbladder, serum, and liver tissues of MAFLD model mice and MAN-treated mice, aiming to clarify the regulatory role of MAN in BA metabolism. Compared with the Normal group, the MAFLD model group showed a significant increase in TBA levels in the gallbladder (Figure 3A), serum (Figure 3B), and liver (Figure 3C). Notably, following MAN intervention, TBA levels in these tissues were reduced to varying degrees. These results demonstrate that MAN can ameliorate MAFLD-associated metabolic abnormalities by regulating bile acid metabolic dyshomeostasis, thereby providing metabolic-level evidence to support its protective effects against MAFLD, including the reduction of body weight and adipose tissue accumulation.

**FIGURE 4:**
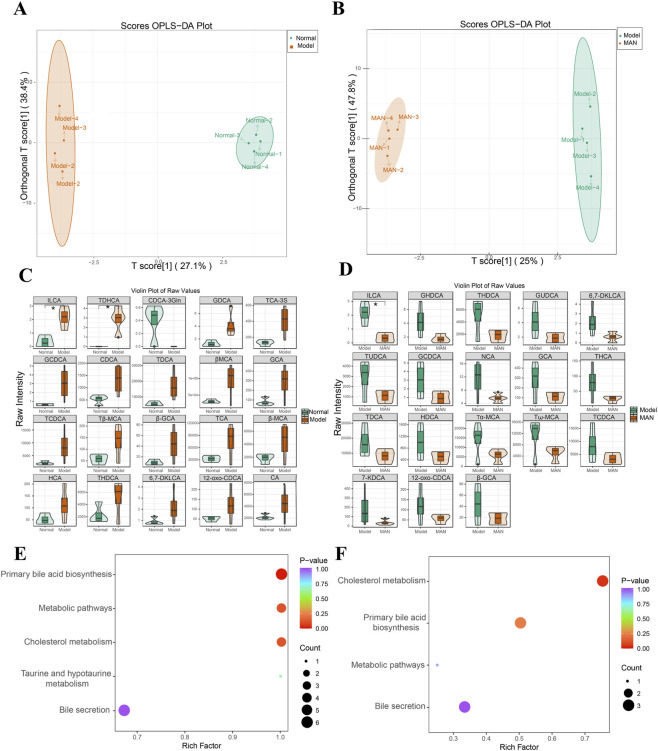
MAN exerts an anti-MAFLD effect by regulating bile acid metabolism. **(A)** and **(B)** principal component analysis. **(C)** and **(D)** Differential metabolite analysis. **(E)** and **(F)** KEGG. ^*^ *p* < 0.05, ^**^ *p* < 0.01, ^***^ *p* < 0.001, ^****^ *p* < 0.0001.

To further elucidate the regulatory effects of MAN on BAs metabolism in MAFLD mice, targeted quantitative analysis of hepatic BAs was performed using mass spectrometry. As shown in Figure 4, the principal component analysis (PCA) score plots (Figures 4A,B) demonstrated clear separations between the Normal and Model groups, as well as between the Model and MAN-treated groups, indicating significant differences in bile acid metabolic profiles among the experimental groups. Differential metabolite analysis (Figures 4C,D) revealed that multiple specific bile acid species were significantly elevated in the MAFLD model group, including isolithocholic acid (ILCA), glycochenodeoxycholic acid (GCDCA), cholic acid-β-glucuronide (βGCA), and taurochenodeoxycholic acid (TCDCA). Notably, MAN intervention markedly reversed the abnormal elevation of these bile acid species, with certain subtypes showing precise and selective regulation. Pathway enrichment analysis (Figures 4E,F) further illustrated the distribution patterns of differential bile acids. In the Model group, altered bile acids were primarily enriched in key pathways such as primary bile acid biosynthesis, bile acid metabolism, cholesterol metabolism, and bile acid secretion. In contrast, in the MAN-treated group, differential bile acids were mainly enriched in cholesterol metabolism, primary bile acid biosynthesis, and bile secretion pathways, suggesting a targeted modulatory effect of MAN on critical metabolic processes. Collectively, these findings indicate that MAN can systematically remodel the bile acid metabolic profile in MAFLD mice by targeting key pathways, including cholesterol metabolism, primary bile acid biosynthesis, and bile secretion, thereby restoring bile acid homeostasis. From the perspective of bile acid metabolic reprogramming, this study provides crucial metabolomics-based evidence supporting the therapeutic effects of MAN against MAFLD and further refines the mechanistic network underlying its action.

### 3.3 MAN Activates the hepatic FXR signaling pathway *in vivo and in vitro*

**FIGURE 5:**
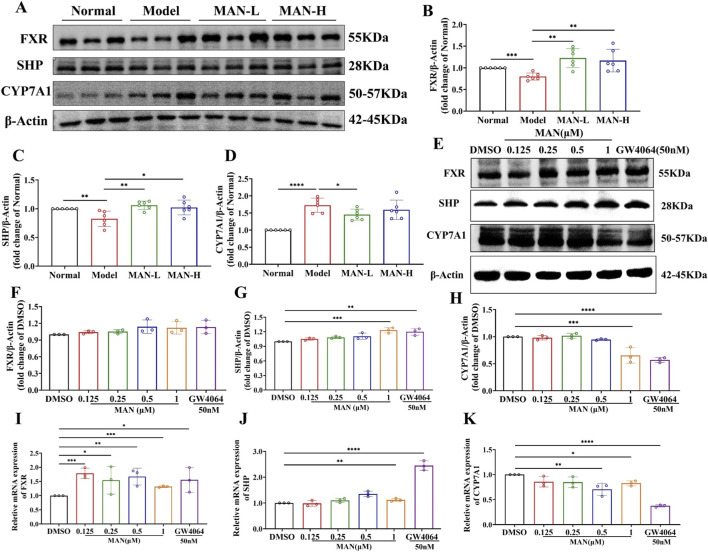
MAN Activates the Hepatic FXR Signaling Pathway *in vivo* and *in vitro*. **(A)** Western blot analysis of FXR, SHP, CYP7A1 protein levels in liver tissue. **(B)** Western blot quantifications of FXR (from A). **(C)** Western blot quantifications of SHP (from A). **(D)** Western blot quantifications of CYP7A1 (from A). **(E)** Western blot analysis of FXR, SHP, CYP7A1 protein levels in HepG2 cells treated with DMSO, MAN (0.125 μM, 0.25 μM, 0.5 μM, 1 μM), or GW4064 (50 nM). **(F)** Western blot quantifications of FXR (from E). **(G)** Western blot quantifications of SHP (from E). **(H)** Western blot quantifications of CYP7A1 (from E). **(I)** qRT-PCR showing mRNA levels of FXR in HepG2 cells treated with DMSO, MAN (0.125 μM, 0.25 μM, 0.5 μM, 1 μM), or GW4064 (50 nM). **(J)** qRT-PCR showing mRNA levels of SHP in HepG2 cells treated with DMSO, MAN (0.125 μM, 0.25 μM, 0.5 μM, 1 μM), or GW4064 (50 nM). **(K)** qRT-PCR showing mRNA levels of CYP7A1 in HepG2 cells treated with DMSO, MAN (0.125 μM, 0.25 μM, 0.5 μM, 1 μM), or GW4064 (50 nM). Data are expressed as the mean ± SD. ^*^ *p* < 0.05, ^**^ *p* < 0.01, ^***^ *p* < 0.001, ^****^ *p* < 0.0001.

Subsequently, we detected the expression levels of proteins associated with the FXR-SHP-CYP7A1 signaling pathway in liver tissues. Western blotting results (Figures 5A–D) showed that compared with the normal group, the protein expressions of FXR (Figures 5A,B) and SHP (Figures 5A,C) in the liver tissues of the MAFLD model group were significantly downregulated, while the protein expression of CYP7A1 (Figures 5A,D) was significantly upregulated. Notably, MAN intervention restored the activated state of the hepatic FXR signaling pathway. To further validate the regulatory effect of MAN on the hepatic FXR signaling pathway, we examined the protein expression and mRNA transcription levels of FXR, SHP and CYP7A1 in HepG2 cells. As shown in (Figures 5E–K), MAN intervention significantly upregulated the protein expression and mRNA transcription levels of FXR and SHP, while downregulating those of CYP7A1. These findings suggest that MAN can regulate the expression of key enzymes involved in BA synthesis by activating the hepatic FXR signaling pathway, thereby ameliorating MAFLD-associated metabolic disorders.

### 3.4 MAN activates the intestinal FXR signaling pathway *in vivo and in vitro*

**FIGURE 6:**
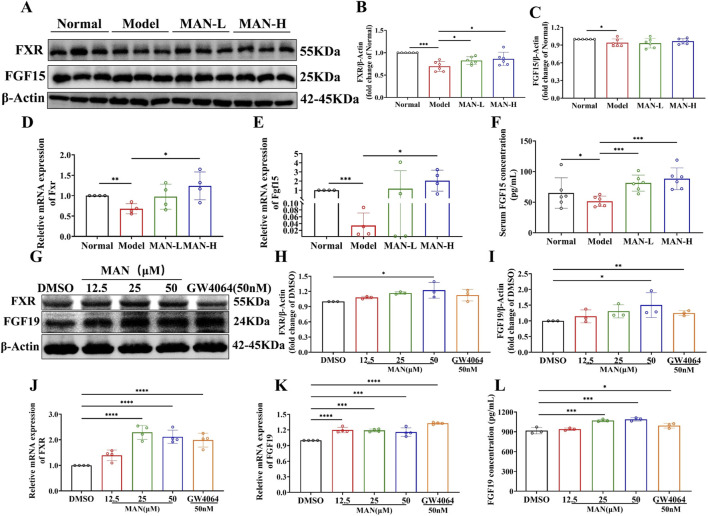
MAN activates the intestinal FXR signaling pathway both *in vivo* and *in vitro*. **(A)** Western blot analysis of FXR, FGF15 protein levels in ileum tissue. **(B)** Western blot quantifications of FXR (from A). **(C)** Western blot quantifications of FGF15 (from A). **(D)** qRT-PCR showing mRNA levels of Fxr in ileum tissue. **(E)** qRT-PCR showing mRNA levels of Fgf15 in ileum tissue. **(F)** Serum FGF15 levels of mice. **(G)** Western blot analysis of FXR, FGF19 protein levels in HT29 cells treated with DMSO, MAN (12.5 μM, 25 μM, 50 μM), or GW4064 (50 nM). **(H)** Western blot quantifications of FXR (from F). **(I)** Western blot quantifications of FGF19 (from F). **(J)** qRT-PCR showing mRNA levels of FXR in HT29 cells treated with DMSO, MAN (12.5 μM, 25 μM, 50 μM), or GW4064 (50 nM). **(K)** qRT-PCR showing mRNA levels of FGF19 in HT29 cells treated with DMSO, MAN (12.5 μM, 25 μM, 50 μM), or GW4064 (50 nM) **(L)** The level of FGF19 in the supernatant of HT29 cells treated with DMSO, MAN (12.5 μM, 25 μM, 50 μM), or GW4064 (50 nM). Data are expressed as the mean ± SD. ^*^ *p* < 0.05, ^**^ *p* < 0.01, ^***^ *p* < 0.001, ^****^ *p* < 0.0001.

Based on the aforementioned experimental results, the intestine serves as the primary absorption site for oral drugs, and the exposure concentration of MAN in the intestine is significantly higher than that in the liver. Accordingly, this study further focused on the regulatory effect of MAN on the intestinal FXR signaling pathway. First, we detected the protein expression and mRNA transcription levels of FXR (Figures 6A,B,D) and FGF15 (Figures 6A,C,E) in the small intestinal tissues of mice (Figures 6A–E). The results showed that in the MAFLD model group, the transcription and protein expression levels of both FXR and FGF15 were significantly downregulated. After MAN intervention, the transcription and protein expression of FXR were markedly increased, and the transcription level of *Fgf15* was also significantly upregulated, while no significant change was observed in FGF15 protein levels. To further clarify the regulatory effect of MAN on the intestinal FXR signaling axis, we measured the protein concentration of FGF15 in mouse serum. The results of the ELISA assay demonstrated that MAN intervention significantly elevated the serum FGF15 level in MAFLD model mice (Figure 6F). Subsequently, we further validated the effect of MAN on the intestinal FXR signaling pathway *in vitro* by detecting the protein expression and mRNA transcription of FXR (Figures 6G,H,J) and FGF19 (Figures 6G,I,K) in HT29 cells. As shown in (Figures 6G–K), MAN intervention dose-dependently increased the protein expression and transcription levels of FXR (Figures 6G,H,J) and FGF19 (Figures 6G,I,K). Additionally, ELISA results demonstrated that MAN intervention significantly elevated FGF19 protein levels in the supernatant of HT29 cells (Figure 6L). Taken together, our findings demonstrate that MAN can alleviate MAFLD-associated metabolic disorders by regulating the intestinal FXR-FGF15/19 signaling axis.

### 3.5 MAN activates the FXR signaling pathway as an FXR agonist

**FIGURE 7:**
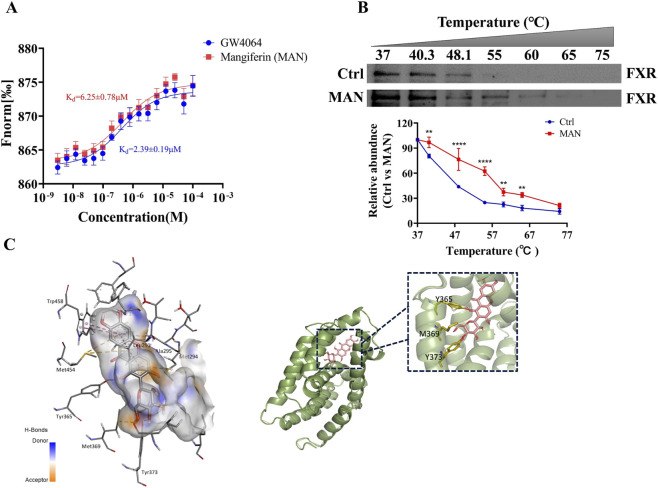
MAN activates the FXR signaling pathway as an FXR agonist. **(A)** Binding affinity of MAN or GW4064 to His-FXR-LBD was detected by MST. **(B)** Cellular CETSA was performed on HT29 at different temperatures. The data were normalized to the mean value of the respective group at 37 °C (n = 3). **(C)** Predicted binding conformation of MAN to FXR and interactions between MAN and FXR. Data are expressed as the mean ± SD. ^*^ *p* < 0.05, ^**^ *p* < 0.01, ^***^ *p* < 0.001, ^****^ *p* < 0.0001.

Subsequently, we validated the direct interaction between MAN and FXR using MST. The results confirmed the binding affinity between the FXR ligand-binding domain (LBD) and MAN, with a dissociation constant (K_d_) of 6.25 ± 0.78 μM (Figure 7A). CETSA was performed on HT29 cells to further corroborate this interaction. Compared with control cells, MAN treatment enhanced the stability of FXR across various temperatures (Figure 7B). Molecular docking analysis was then conducted to investigate the direct binding mode between FXR and MAN. In the crystal structure of FXR (PDB ID: 1OSH), MAN successfully docked into the binding pocket via hydrogen bonds with residues Y365, M369, and Y373, which located on both the upper and lower sides of the pocket (Figure 7C).

**FIGURE 8:**
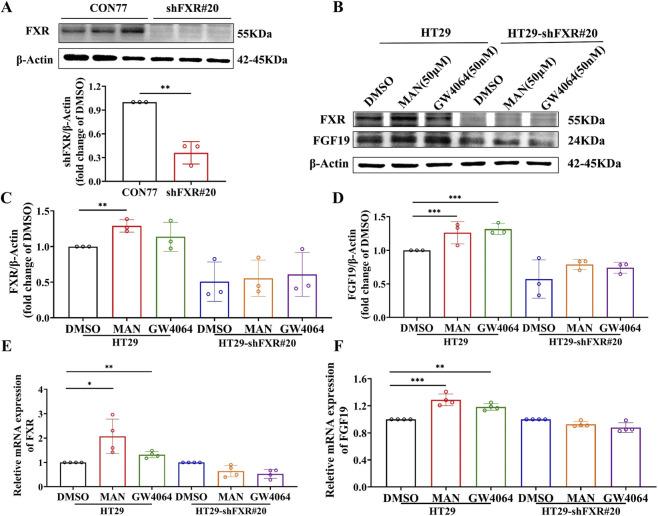
ShFXR inhibits the activation of intestinal FXR signaling pathway by MAN. **(A)** Western blot bands (top) and quantitative analysis (bottom) of FXR protein level in HT29 cells transfected with control (CON77) or shFXR (#20) vectors. **(B)** Western blot bands of FXR and FGF19 in wild-type HT29 cells and shFXR-transfected HT29 cells (HT29-shFXR#20) treated with DMSO, MAN (50 μM), or GW4064 (50 nM) **(C)** Western blot quantifications of FXR (from B). **(D)** Western blot quantifications of FGF19 (from B). **(E)** qRT-PCR showing mRNA levels of FXR in wild-type HT29 cells and HT29-shFXR#20 cells treated with DMSO, MAN (50 μM), or GW4064 (50 nM). **(F)** qRT-PCR showing mRNA levels of FGF19 in wild-type HT29 cells and HT29-shFXR#20 cells treated with DMSO, MAN (50 μM), or GW4064 (50 nM). Data are expressed as the mean ± SD. ^*^ *p* < 0.05, ^**^ *p* < 0.01, ^***^ *p* < 0.001, ^****^ *p* < 0.0001.

To further verify the targeted interaction between MAN and FXR, we knocked down endogenous FXR expression using shRNA. First, shRNA effectively downregulated the protein expression level of FXR (Figure 8A). Western blot results showed that MAN intervention significantly upregulated the protein expression of FXR and FGF19 in normal HT29 cells (Figures 8B–D); qRT-PCR analysis further confirmed that MAN markedly increased the transcription levels of *FXR* and *FGF19* (Figures 8E,F). However, in FXR-knockdown HT29 cells (shFXR-HT29), both Western blot and qRT-PCR results demonstrated that shFXR completely blocked the MAN-induced upregulation of FXR at both the protein and transcription levels, while abrogating its regulatory effect on FGF19 (Figures 8B–F). In summary, these findings demonstrate that MAN functions as an FXR agonist. By directly activating the FXR signaling pathway, MAN provides critical molecular mechanistic support for ameliorating MAFLD-associated metabolic disorders, further confirming FXR as the core target underlying anti-MAFLD efficacy of MAN.

### 3.6 Modulating effects of MAN on gut microbial composition

**FIGURE 9:**
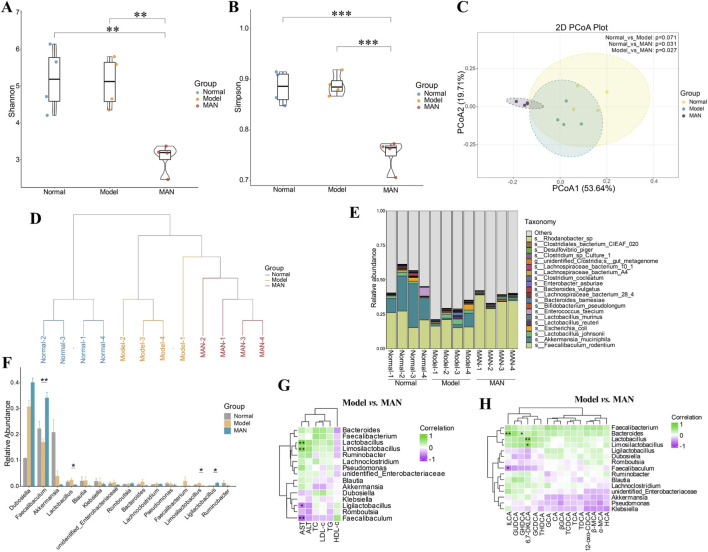
Modulating effects of MAN on gut microbial composition. α-diversity in the **(A)** Shannon index and **(B)** Simpson index. **(C)** Principal component analysis. **(D)** Phylogenetic clustering analysis (UPGMA). **(E)** Relative abundance at the Genus level. **(F)** Microorganisms with differential abundance at the genus level. **(G)** Correlation analysis between serum metabolic parameters and the top 15 differentially abundant genera identified in **(F)** (Model vs. MAN). **(H)** Correlation analysis between differential metabolites and the top 15 genera identified in **(F)** (Model vs. MAN). Data are expressed as the mean ± SD. ^*^ *p* < 0.05, ^**^ *p* < 0.01, ^***^ *p* < 0.001, ^****^ *p* < 0.0001.

Gut microbiota has been increasingly recognized as an important contributor to the development and progression of MAFLD. To investigate the regulatory effect of MAN on the gut microbiota in MAFLD, 16S rRNA sequencing was performed on fecal samples from the Normal, model, and MAN groups. The results showed that, compared with the Normal group, the model group exhibited altered gut microbial α-diversity, while MAN intervention further changed the diversity indices (Figures 9A,B). Specifically, the Shannon (Figure 9A) and Simpson (Figure 9B) indices were significantly decreased in the MAN-treated group, suggesting that MAN may reshape the gut microbial structure through the selective enrichment of dominant bacterial taxa. PCA further revealed a clear separation among the three groups, indicating significant differences in microbial community structure, with the MAN group distinctly separated from the model group (Figure 9C). Consistently, phylogenetic clustering analysis demonstrated that samples from different groups formed relatively independent clusters, further supporting that MAN modulates the overall gut microbial composition in MAFLD mice (Figure 9D).

At the species level, significant differences in microbial taxa were observed among the groups (Figure 9E). Notably, *Lactobacillus murinus* and *Faecalibaculum rodentium* were significantly enriched following MAN treatment. At the genus level, MAN markedly increased the relative abundances of *Faecalibaculum* and *Ligilactobacillus*, while decreasing those of *Lactobacillus* and *Limosilactobacillus* compared with the model group (Figure 9F), suggesting that MAN may alleviate gut microbial dysbiosis in MAFLD mice by modulating key bacterial taxa. To further elucidate the functional interplay between the gut microbiome and host metabolism, we performed a correlation analysis focusing on the top 15 differentially abundant genera. As depicted in Figure 9G, *Lactobacillus* and *Limosilactobacillus* exhibited positive correlations with key serum metabolic and hepatic injury markers, including TG, TC, LDL-C, AST, and ALT. Conversely, *Faecalibaculum* and *Ligilactobacillus* were negatively correlated with AST, ALT, and TG. These contrasting patterns suggest that these specific bacterial taxa are deeply implicated in the progression of lipid metabolic disorders and liver injury. Furthermore, correlation analysis between these top 15 genera and targeted bile acids demonstrated that *Lactobacillus*, *Bacteroides*, and *Limosilactobacillus* were positively correlated with 6,7-DKLCA, ILCA, GHDCA, and GUDCA, while *Faecalibaculum* showed negative correlations with these bile acids (Figure 9H). Notably, ILCA and 6,7-DKLCA are secondary bile acids, whereas GHDCA and GUDCA are conjugated bile acids, which are often elevated under MAFLD conditions and closely associated with liver injury and lipid metabolic disorders. Taken together, these findings suggest that bacterial taxa positively associated with abnormal bile acids (e.g., *Lactobacillus* and *Limosilactobacillus*) were also positively correlated with dyslipidemia and liver injury markers, whereas bacteria negatively correlated with these bile acids (e.g., *Faecalibaculum*) were associated with improved metabolic status. These results indicate that MAN may improve MAFLD-associated metabolic disturbances by remodeling the gut microbiota, thereby modulating bile acid metabolism and subsequently improving lipid metabolism and liver function.

## 4 Discussion

MAFLD, characterized by hepatic lipid accumulation, has reached a prevalence of 28%–40% in the Asia-Pacific region and continues to rise (Chiang, 2013). Currently, there is a lack of safe and effective pharmacological treatments, highlighting the urgent need for novel therapeutic agents. Given the central role of FXR in BAs metabolism and MAFLD progression, FXR-targeted interventions have become a focal point in MAFLD research. Previous studies have demonstrated that Hyperoside alleviates high-fat diet-induced NAFLD via FXR activation (Wang et al., 2026), and Bruceine D selectively modulates BA synthesis through the FXR-SHP pathway to mitigate cholestatic liver injury (Fu et al., 2026). However, the precise mechanisms underlying these natural products’ modulation of FXR remain unclear. In our study, MST, CETSA, and molecular docking analyses revealed that MAN directly activates FXR, with Y365, M369, and Y373 identified as potential binding sites. This activation was further validated in shRNA-mediated FXR knockdown experiments. Notably, MST showed a moderate binding affinity of MAN to FXR (Kd = 6.25 ± 0.78 μM), lower than that of the potent synthetic agonist GW4064, but this may confer a therapeutic advantage. Excessive receptor affinity, such as that of obeticholic acid (OCA), can induce persistent FXR activation and associated adverse effects including pruritus, dyslipidemia, and dramatic fluctuations in the BA pool (Russell, 2009; Tian et al., 2022). Furthermore, the long-term use of traditional metabolic drugs presents additional clinical challenges; for instance, statins may interfere with glucose metabolism, elevating the risk of incident diabetes or exacerbating glycemic control in patients with pre-existing diabetes (Mansi et al., 2021), while the use of thiazolidinediones (such as rosiglitazone and pioglitazone) frequently leads to undesirable weight gain (Paschoal et al., 2020). Collectively, these limitations underscore the therapeutic advantage of MAN, whose moderate affinity and natural origin position it as a potentially safer and highly promising candidate for MAFLD management.

Beyond direct receptor activation, gut microbiota-mediated BA remodeling also plays a pivotal role in MAFLD progression. Modulation of the gut microbial ecosystem has emerged as an important strategy for natural product-based MAFLD interventions (Tang et al., 2026). For example, Bao et al. reported that Stevia root polysaccharides (SRRP) ameliorate HFD-induced NAFLD by enriching beneficial microbes such as *Akkermansia* and suppressing harmful bacteria (Sinha and Kumar, 2026), while Liu et al. demonstrated that torularhodin mitigates NAFLD in male mice via gut microbiota regulation (Liu et al., 2025). Additionally, Huang et al. suggested that Oroxin B improves MAFLD by alleviating gut microbiota dysbiosis in a high-fat diet-induced rat model (Huang Y. et al., 2023). In this study, MAN intervention significantly reduced the relative abundance of *Lactobacillus* and *Limosilactobacillus*, which positively correlated with aberrant BAs including 6,7-DKLCA, ILCA, GHDCA, and GUDCA, as well as dyslipidemia and hepatic injury markers. Conversely, the abundance of *Faecalibaculum* increased following MAN treatment and negatively correlated with these BAs, concomitant with improved metabolic outcomes. Previous studies have reported that *Ligilactobacillus* efficiently eliminates acetaldehyde accumulation and reverses key indicators of liver fibrosis in HFHFD-induced models (Tang et al., 2026). Similarly, *Limosilactobacillus mucosae* FZJTZ26M3 has been shown to prevent NAFLD through the modulation of lipid metabolism and gut microbiota dysbiosis (Dang et al., 2024). In contrast, the abnormal expansion of certain *Lactobacillus* species under HFD conditions has been linked to BAs metabolic disorders, suggesting that their reduction may help restore BAs homeostasis (Wei et al., 2026). These findings elucidate the molecular basis by which MAN regulates MAFLD via the “microbiota-BAs axis” and provide strong experimental and theoretical support for developing microbiota-targeted, natural product-based therapies.

MAFLD pathogenesis involves intertwined processes of inflammation, oxidative stress, and dysregulated glucose–lipid metabolism, making single-target interventions insufficient (Huang X. et al., 2023). The multi-component and multi-target characteristics of natural products are particularly advantageous in this context. Consistent with our previous findings, MAN activates AMPK signaling to improve glucose transport and lipid metabolism, reducing hepatic steatosis, while simultaneously suppressing NLRP3 inflammasome activation, attenuating proinflammatory cytokine release, and alleviating liver inflammation (Yong et al., 2021). Our study demonstrates that MAN exerts anti-MAFLD effects through coordinated activation of the FXR pathway and modulation of gut microbial homeostasis to maintain BA balance, intervening at multiple critical nodes in disease progression. Collectively, MAN, as a multi-target natural bioactive compound, exhibits unique advantages in MAFLD prevention and treatment, providing a theoretical basis and direction for the development of safe and effective metabolic disease interventions. Future studies will focus on elucidating the structure-activity relationship (SAR) of MAN-FXR interaction and further clarifying the precise mechanistic linkages within the “gut microbiota-BAs-FXR” axis, to guide formulation optimization and clinical translation of MAN.

## 5 Conclusion

This study demonstrates that MAN, as a natural FXR agonist, effectively regulates bile acid metabolism through direct activation of the FXR signaling pathway, thereby ameliorating glucose and lipid metabolic disorders. In addition, MAN can remodel the gut microbiota composition, indirectly modulating bile acid metabolism and consequently alleviating hepatic steatosis and liver injury. These findings indicate that MAN exerts protective effects against MAFLD through a dual mechanism involving FXR signaling activation and gut microbiota modulation, providing important theoretical support for its development as a potential therapeutic agent for the prevention and treatment of MAFLD.

## Data Availability

The raw data supporting the conclusions of this article will be made available by the authors, without undue reservation.
